# Interleukin-6: Molecule in the Intersection of Cancer, Ageing and COVID-19

**DOI:** 10.3390/ijms21217937

**Published:** 2020-10-26

**Authors:** Jan Brábek, Milan Jakubek, Fréderic Vellieux, Jiří Novotný, Michal Kolář, Lukáš Lacina, Pavol Szabo, Karolína Strnadová, Daniel Rösel, Barbora Dvořánková, Karel Smetana

**Affiliations:** 1 Department of Cell Biology, Faculty of Science, Charles University, 120 00 Prague 2, Czech Republic; jan.brabek@natur.cuni.cz (J.B.); Daniel.Rosel@natur.cuni.cz (D.R.); 2 BIOCEV, Faculty of Science, Charles University, 252 50 Vestec, Czech Republic; 3 Centre for Tumour Ecology, First Faculty of Medicine, Charles University, 120 00 Prague 2, Czech Republic; Milan.Jakubek@lf1.cuni.cz (M.J.); Frederic.Vellieux@lf1.cuni.cz (F.V.); Jiri.Novotny@img.cas.cz (J.N.); Michal.Kolar@img.cas.cz (M.K.); Lukas.Lacina@lf1.cuni.cz (L.L.); Karolina.Strnadova@lf1.cuni.cz (K.S.); Barbora.Dvorankova@lf1.cuni.cz (B.D.); 4 Department of Paediatrics and Adolescent Medicine, First Faculty of Medicine, Charles University and General University Hospital, 120 00 Prague, Czech Republic; 5 BIOCEV, First Faculty of Medicine, Charles University, 252 50 Vestec, Czech Republic; 6 Department of Analytical Chemistry, University of Chemistry and Technology Prague, 166 28 Praha 6, Czech Republic; 7 Laboratory of Genomics and Bioinformatics, Institute of Molecular Genetics, Czech Academy of Sciences, 140 00 Prague 4, Czech Republic; 8 Institute of Anatomy, Fist Faculty of Medicine, Charles University, 120 00 Prague 2, Czech Republic; szabopavol@gmail.com; 9 Department of Dermatovenereology, First Faculty of Medicine, Charles University and General University Hospital, 120 00 Prague 2, Czech Republic

**Keywords:** tumour microenvironment, cancer ecosystem, ageing, COVID-19, IL-6, cytokine storm, cytokine, cancer-associated fibroblasts

## Abstract

Interleukin-6 (IL-6) is a cytokine with multifaceted effects playing a remarkable role in the initiation of the immune response. The increased level of this cytokine in the elderly seems to be associated with the chronic inflammatory setting of the microenvironment in aged individuals. IL-6 also represents one of the main signals in communication between cancer cells and their non-malignant neighbours within the tumour niche. IL-6 also participates in the development of a premetastatic niche and in the adjustment of the metabolism in terminal-stage patients suffering from a malignant disease. IL-6 is a fundamental factor of the cytokine storm in patients with severe COVID-19, where it is responsible for the fatal outcome of the disease. A better understanding of the role of IL-6 under physiological as well as pathological conditions and the preparation of new strategies for the therapeutic control of the IL-6 axis may help to manage the problems associated with the elderly, cancer, and serious viral infections.

## 1. Introduction

Interleukin-6 (IL-6) is a bioactive protein known under numerous synonyms (Table 1). It is a cytokine of a pro-inflammatory nature, and it can be produced by various cell types of the immune system as well as by some nonimmune cells, including fibroblasts. Regarding the anatomical distribution of Il-6, it was identified in the lungs, urinary bladder, adipose tissue, muscles, vermiform appendix, etc. (The Human Protein Atlas, [1]). 

The main cell types acting as producers of IL-6 are shortlisted in Table 2.

IL-6 is recognised by its transmembrane receptor (IL-6R), which forms a complex with glycoprotein 130 (gp130). This receptor has tyrosine kinase activity and activates signal transducer and activator of transcription 3 (STAT3) via phosphorylation. On the other hand, the extracellular portion of IL-6R can be cleaved from the intramembranous domain of the receptor by membrane protease ADAM-17. Soluble IL-6R without tyrosine kinase activity interacts with gp130 outside the cell and forms a complex of IL-6, soluble IL-6R and gp130, which is docked back to the cell membrane [20]. This arrangement of the IL-6–IL-6R axis can be functionally variable when the actual function of IL-6 signalling is dependent on the type of cell and the type of interacting receptor. While the interaction of IL-6 with transmembrane IL-6R and gp130 participates in anti-inflammatory pro-cancerogenic signalling, the interaction of IL-6 with soluble IL-6R and gp130 stimulates inflammation [20]. 

In this review, we aim to highlight the molecular similarity between apparently distinct phenomena and their mechanisms such as physiological ageing, formation of the cancer niche ecosystem and severe inflammatory conditions, including viral infections such as COVID-19. In all of them, we can invariably observe deregulation of the IL-6–IL-6R axis. Therefore, our more in-depth insight into the IL-6 function in the context of ageing, tumourigenesis and infections may bring new therapeutic strategies for the treatment of age-related disorders, cancer and transmissible, e.g., viral, diseases.

## 2. Physiological Functions of IL-6 

The family of IL-6-related proteins consists of members with remarkable and distinct biological activities that are structurally similar to IL-6, such as IL-11, IL-31, cardiotrophin-1, ciliary neurotrophic factor (CNTF), cardiotrophin-like cytokine (CLC), granulocyte colony-stimulating factor (G-CSF), leptin, leukaemia inhibitory factor (LIF), neuropoietin, and oncostatin [21]. This cytokine family is defined by sharing common IL-6 family signalling receptor gp130 more than by any structural homology of its members. It is therefore not surprising that the IL-6 family cytokines not only display partially overlapping, but also, more significantly, very different biological activities [22].

IL-6 knockout mice are available for research purposes [23]. Interestingly, their embryonic and foetal development is not hampered, and knockout animals do not have any apparent developmental abnormalities. On the other hand, these mouse strains were highly susceptible to several pathogens, and they failed to generate acute-phase responses [24]. 

IL-6 contributes to the host defence by stimulation of the acute phase immune response, including elevation of body temperature [25]. In this context, IL-6 positively influences the maturation of B lymphocytes and cytotoxic T lymphocytes [26,27]. In the same motion, IL-6 deficiency in an experimental model leads to protection against triggering autoimmune encephalomyelitis [28]

IL-6 also belongs to the family of myokines such as IL-8, IL-15, irisin, myostatin, fibroblast growth factor (FGF)21, leukemia inhibitory factor (LIF), brain-derived neurotrophic factor (BDNF), and insulin like growth factor-1 (IGF-1) that influence the function of skeletal muscle with metabolic impacts on the whole organism [16], namely by interaction with adipocytes and factors produced by these cells [29]. In knockout mice, surviving animals had reduced age-related obesity development [30]. 

The role of IL-6 in the bone metabolism was also confirmed by the stimulation of osteoclast activity [31]. This is in good agreement with the observed protection against the bone loss after ovariectomy in a mouse knockout model [32]. 

These few examples demonstrate the complex and multifaceted role of IL-6 both in physiological and pathological conditions in the human body.

## 3. IL-6 and Ageing

### 3.1. “Inflammaging” as a Developmentally Controlled Process 

In the last seventy years, the life expectancy of citizens dramatically increased in many countries across the world. In a number of developed countries, it now reaches around 80 years of age. This represents an increase in life expectancy from the beginning of the 20th century by approximately 30 years. Unfortunately, this trend is associated with numerous age-related phenomena such as cardiovascular diseases, cognitive function impairment, sarcopoenia, metabolic disorders, cachexia, and also an increased incidence of cancer [33]. “Adding years to life and life to years” [34] has become an appealing manifesto of health care-providing authorities in recent times. It urges in-depth insights into the mechanisms typical of healthy longevity. 

Even in the absence of any disease, chronologically aged cells differ from juvenile cells. Bioinformatic analysis of events associated with the ageing of tissues and organs in otherwise healthy seniors highlighted specific developmentally regulated mechanisms. Surprisingly, age-induced changes are typical of inflammation [35]. This finding correlates well with an increasing number of neutrophils—cells of innate immunity. On the other hand, lymphocytes—the principal cells of adaptive immunity—are significantly reduced during ageing [36]. One of the typical humoral markers of inflammation, namely in the early stage, is IL-6. The serum concentration of IL-6 increases during ageing, and it is independent of ethnicity [36,37,38]. The moderately elevated serum concentration of IL-6 in aged people plays a significant role in functional impairment, including low locomotion, cognitive and mental functions, and depression [39]. The highly elevated level of this cytokine can even predict increased mortality in very old individuals [40]. On the other hand, a low level of IL-6 representing lower “inflammaging” was typical of successful centenarians [41]. 

**Figure 1:**
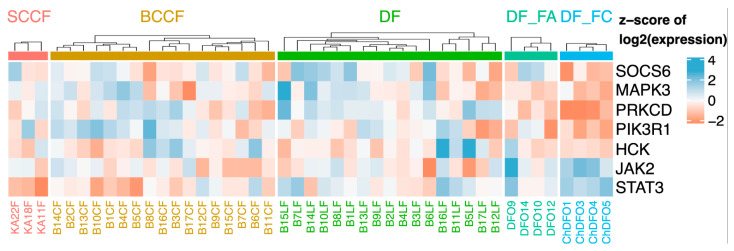
Multiple genes of the interleukin-6 (IL-6) signalling pathway display gradual changes in transcription activity, differing among facial dermal fibroblasts from children (DF_FC), healthy adults (DF_FA), photodamaged dermal fibroblasts (DF) of patients suffering from basal cell carcinoma, and cancer-associated fibroblasts (CAFs) from basal cell carcinomas (BCCF) and cutaneous squamous cell carcinomas (SCCF).

Ageing in itself is not a disease [42]. However, it is a condition that allows or induces the emergence of some diseases. Even healthy senescent tissue, devoid of clinically apparent disease, exerts some alarming molecular features. This might be exemplified by the study of the expression profiles of the dermal fibroblasts isolated from very old donors. These fibroblasts demonstrated a remarkable similarity to cancer-associated fibroblasts (CAFs), including a high expression of mRNA encoding IL-6 [43]. Indeed, transcriptional profiling of facial dermal fibroblasts from children, healthy adults, photodamaged dermal fibroblasts of patients suffering from basal cell carcinoma, and CAFs directly from basal and cutaneous squamous cell carcinomas revealed striking similarities in the expression of downstream components of the IL-6 signalling pathway [44] between aged fibroblasts and CAFs. Notably, Janus kinase 2 (JAK2) and signal transducer and activator of transcription 3 (STAT3) displayed a clear rising trend from very low activity in child fibroblasts, through to intermediate activity in photoaged dermal fibroblasts, and then to elevated activity in CAFs (Figure 1), indicating an increasing degree of “inflammaging” 

As stated above, ageing is not a disease. However, ageing and disease are frequently tightly associated. As noted by the WHO, health shall not be understood as an absence of disease [34]. Hence, the tie of ageing and disease is sometimes so close that it is obviously challenging at this level to draw a sharp demarcation line separating “healthy yet aged tissue” from already precancerous or even cancerous tissue. 

Our understanding of “inflammaging” and the significance of elevated IL-6, C-reactive protein (CRP) and tumour necrosis factor (TNF)-α concentrations is not yet complete. The role of a developmentally programmed genetic process is hypothesised. Still, other processes, such as chronic viral infection (cytomegalovirus), a high volume of visceral fat, altered gut permeability, ineffective immune response in the elderly and the accumulation of senescent cells in the body may also be responsible for “inflammaging” [45]

### 3.2. Non-Steroid Anti-Inflammatory Drugs as “Inflammaging” Therapy in Ageing

Upon broadly accepted medical advice, the aged population frequently uses low-dose acetylsalicylic acid (ASA). It acts as prevention of thromboembolism and cardiovascular diseases, mainly because of its anticlotting effect. It was observed in many studies that this prophylactic application also has a significant anti-cancer effect, at least for cancer of the prostate, lung, colorectum, ovary, uterus, and stomach [46,47,48]. ASA is a member of the non-steroid anti-inflammatory drug family. The molecular mechanism of ASA activity is well known, and it is explained by irreversible acetylation of cyclooxygenase (COX) enzymes, resulting in the anti-inflammatory effect. It seems that the anti-cancer effect of non-steroid anti-inflammatory drugs is not strictly dependent on the abovementioned molecular mechanism. It was confirmed that other non-steroid anti-inflammatory drugs affecting other molecular mechanisms are also beneficial in cancer prevention, as exemplified in breast cancer [49,50]. ASA and other non-steroid anti-inflammatory drugs can successfully reduce “inflammaging” in the tissue/organ microenvironment. Such inhibition can adversely impact the cancer ecosystem and thus consequently inhibit the probability of successful cancer initiation and growth in this niche. In the context of this article, it should be mentioned that these substances reduce the actual levels of IL-6 and TNF-α, factors known for their supporting role in tumour growth and spread, as discussed above [49,50]. Moreover, ASA has a direct effect on the production of IL-6 by adipocytes, and so it has an indirect effect on cancer [51]. ASA also stimulates apoptosis in cancer cells [52]. Its effect on blood platelets has also demonstrated the role of non-steroid antirheumatic drugs in cancer. The anti-platelet activity of low doses of ASA in combination with COX in suppressing tumourigenesis was clearly established [53]. However, ASA and other non-steroid anti-inflammatory drugs display side effects, namely in the gastrointestinal system, where they induce gastric erosions, ulcerations, and bleeding. According to the U.S. Preventive Services Task Force Recommendation [54], the risk of bleeding is minimal after low-dose use, and the benefits, including colorectal cancer prevention, prevail. The data about ASA and other non-steroid anti-inflammatory drugs in cancer prevention by modulation of the cancer microenvironment may be an inspiration for the development of novel preventive strategies for cancer incidence reduction in the elderly. 

### 3.3. Summary of the Role in IL-6 in Ageing

Ageing is associated with a proclivity to inflammation. At the cellular level, accumulating evidence shows that senescent cells may have deleterious effects on the tissue microenvironment [55]. The elevation of IL-6 notably accompanies this developmental programme of ageing. Apart from its orderly physiological functions, the IL-6 cytokine plays a fundamental role in the intercellular communication between various cells across tissues harbouring a potentially cancerogenic mutational burden. IL-6 acts as a key messenger between cancerous and non-cancerous cell populations at the tumour site. It strengthens their local interactions, but it also has a prominent systemic effect after leakage to the circulation. Terminal stages of disease in the elderly and malignant diseases share outstanding similarities. Both in ageing-related and cancer-induced cachexia, IL-6 alone, or in combination with other factors, plays a critical role [56]. Evidence suggests that long-term prophylactic systemic therapy by recently available non-steroid anti-inflammatory drugs in the elderly can be beneficial for these individuals. Apart from improved cardiovascular outcomes, this therapy can lead to a reduction in the incidence of malignant tumours in aged patients. However, even this therapy should be carefully individualised. It was revealed in recent years that the ASA effects on several condition outcomes, including cancer, also showed interactions particularly with body mass [57]. Therefore, the simplistic “one-dose-fits-all” strategy of prevention is unlikely to be optimal.

## 4. IL-6 and Chronic Inflammatory Diseases

Human pathology and clinical medicine describe a plethora of chronic inflammatory systemic diseases [58]. In recent years, many of these conditions have been classified as autoimmune disorders, namely rheumatoid arthritis, systemic lupus erythematosus, and multiple sclerosis, and should be associated with tumour formation. In agreement with the topic of this article, participation of chronic inflammation in experimentally induced inflammatory bowel disease induces tissue fibrosis, which promotes cancer in the treated animals [59]. Because of the critical role of IL-6 in the control of inflammation, it is not surprising that this cytokine is essential in the chronic, frequently autoimmune inflammations listed above. Notably, in rheumatoid arthritis, the blocking of the IL-6–IL-6R axis can be successfully controlled in clinical practice, for example, by tocilizumab [60]. However important, all these conditions originating from the immune cell aggression against the organism, in principle, represent very different issues that are relatively remote to the scope of our review. Therefore, we decided not to follow this aspect and focus primarily on the oncological and developmental implications. 

## 5. Cancer and IL-6

### 5.1. Cancer as a Complex Tissue/Organ

The incidence of malignant tumours in humans is significantly increasing. This phenomenon seems to be associated with population ageing in developed countries, where it is traditionally attributed to advanced medical care. We may expect that each third or even every other citizen is at risk of encountering cancer in the course of his or her life [61]. Therefore, all knowledge improving our understanding of cancer biology is critically important because it can establish a basis for new therapeutic strategies. The scientific interest, as well as therapeutic efforts, have primarily focused on cancer cells. This population is usually widely genetically altered [62] because of the attenuation of the gene repair machinery in the elderly [63]. This concept was successful many times and allowed for even highly efficient, personalised treatment, as exemplified in everyday practice on the case of, e.g., BRAF-mutated melanoma. However, a tumour is a complex tissue and contains highly important yet non-cancerous components, usually described as the stroma. In a broader view, the tumour can be described using the terminology and principles of classical ecology. This approach allows for the identification of some previously neglected functional interactions. In this concept, the tumour cells reside in a suitable niche that can support them via nutrients and oxygen, and that also provides protection against predators such as anti-cancer immunity [64]. In parallel, it is known that normal adult stem cells also require a similar specific environment for their life-long stemness maintenance [65]. This suggests an intriguing similarity between some aspects of cancer and regenerative biology. It is not surprising that cancer was tentatively compared to chronic wounds [66], and remarkable similarities between the wound repair mechanisms and cancer were indeed identified based on the molecular architecture of the healing process [67]. 

**Figure 2:**
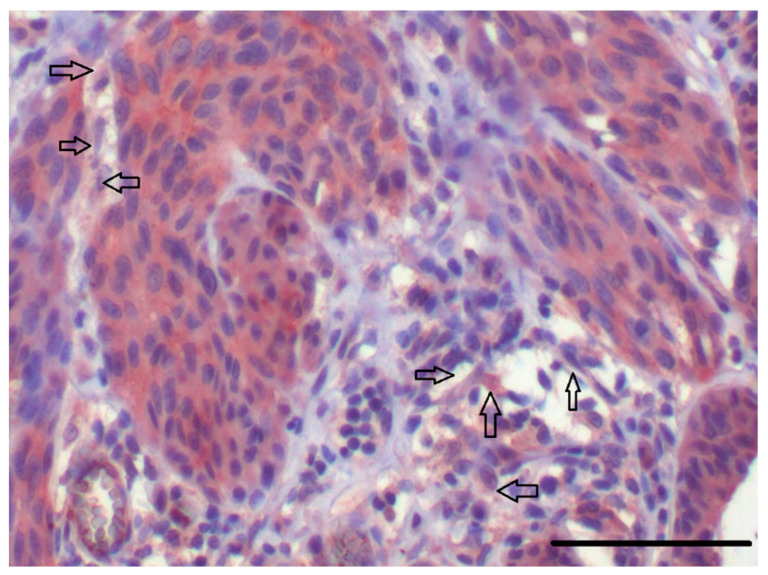
Positive immunohistochemical detection of IL-6 in human cutaneous malignant melanoma. Nests of melanoma cells are highly positive for IL-6 (in brown). Stromal cells, including representatives of CAFs (arrows), are also somewhat positive in this staining. The bar is 100 μm.

Except for cancer cells, the cancer ecosystem contains cancer-associated fibroblasts (CAFs) and infiltrating immune cells ((natural killer (NK) cells, Treg lymphocytes, CD8+ T lymphocytes, tumour-associated macrophages, myeloid-derived immunosuppressive cells, etc. [68]) (Figure 2). From this point of view, a tumour, for example, cancer of the breast, can be seen as a parallel of a specific organ. It requires highly orchestrated regulation that improves the tumour growth and consequently allows its spread [43]. It is critically important to identify individual components of the tumour, but it is even more important to be able to identify all the interactions that they are undergoing. 

### 5.2. CAFs as Producers of IL-6

**Figure 3:**
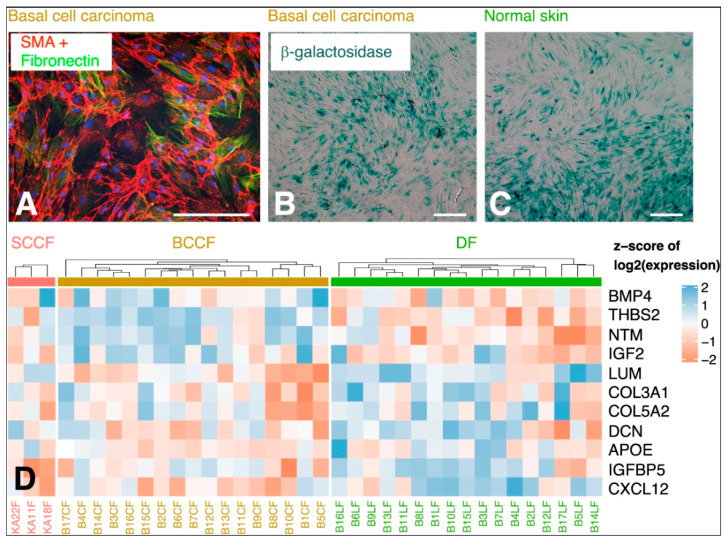
Cultured cancer-associated fibroblasts from basal cell carcinoma and normal skin. Part of fibroblasts isolated from the tumour exhibit α-smooth muscle actin (SMA; green signal). All cells produce fibronectin (red signal). Nuclei were counterstained with 4’,6-diamidino-2-phenylindole (DAPI; blue signal) (**A**). Cultured normal dermal fibroblasts (DF) from the face of an aged donor (**B**) and CAFs from basal cell carcinoma (BCCF) from the face of the same donor (**C**) contain a very high proportion of senescent fibroblasts positive for senescence-associated acid β-galactosidase. The bar is 100 μm. While the senescent phenotype is present in both fibroblast groups, the cells differ in gene expression of several senescence-associated secretory phenotype (SASP) markers (**D**). The same genes are strongly expressed in CAFs from cutaneous squamous cell carcinoma (SCCF).

The cancer ecosystem is quite uniform in different types of tumours. Apart from the cancer cells and immune cells, it contains large numbers of fibroblasts, the CAFs. These cells seem to be important in the control of coordination of the whole cancer ecosystem [69,70,71]. However, these cells differ from normal tissue fibroblasts in many aspects. Functionally, CAFs are highly activated, and they frequently express α-smooth muscle actin in the majority of tumours (Figure 3A). No unique or universal marker of CAFs has been described thus far. CAFs express several characteristic proteins such as fibroblast-activating protein (FAP), tenascin-C, periostin, Thy-1, podoplanin, and caveolin-1 [72]. In practice, we usually have to rely on a combination of several markers. Therefore, distinguishing them exactly from normal fibroblasts, namely in tissue sections, is not simple. In research practice, several markers should be detected to estimate their quantity and activity. 

The origin of CAFs is not entirely clear. It is most likely that CAFs are formed from the local mesenchyme, namely fibroblasts. Similarly, their origin in other mesenchymal cell populations, e.g., adipocytes, pericytes, or endothelial cells, was also hypothesised [71].

Alternatively, CAFs may originate from the bone marrow mesenchymal stem cells chemoattracted to the tumour site, which is less likely via epithelial-to-mesenchymal transition [73,74]. 

It is known that CAFs are formed from their precursors by factors such as transforming growth factor β (TGF-β)1/3, inflammatory signals such as IL-6, and proteins such as platelet-derived growth factor (PDGF), FGF and galectin-1. The damage to DNA by previous chemo/actinotherapy and reactive oxygen species (ROS) can also enhance CAF formation [71,75,76]. 

CAFs are not a homogeneous population, and they can be further stratified to several subgroups. Such clustering would slightly differ according to the type of tumours and the stage of the disease. However, it can be concluded that part of CAFs usually produce the extracellular matrix (Figure 3) and others secrete bioactive proteins that influence the biological properties of cancer cells [72]. CAFs are frequently characterised by their senescence-associated secretory phenotype (SASP) [55,77]. This feature is also seen in aged fibroblasts [78]. Despite a general similarity in the expression of SASP components [79], several genes of the SASP signature differ in their expression between CAFs from cutaneous squamous cell carcinoma, basal cell carcinoma, and photodamaged facial fibroblasts of the same patient (Figure 3D).

CAFs from basal or squamous cell carcinoma (all from the head and neck) are bioactive in normal epithelial cells, where they control their low differentiation status [69,70]. Interestingly, CAFs from the basal cell carcinoma, squamous cell carcinoma, breast cancer, and melanoma significantly influenced the phenotype of the breast cancer cell line to the more aggressive appearance close to the breast cancer stem cells [80], which underlines the non-specific character of the crosstalk between the cancer cells and CAFs within the cancer ecosystem. CAFs prepared from malignant cutaneous melanoma significantly improve in vitro migration of glioblastoma cells [81]. However, CAFs are also able to influence the phenotype of fibroblasts in their vicinity, which consequently acquire the phenotype and differentiation plasticity of mesenchymal stem cells [82]. This observation may help to explain the observation of ectopic cartilage or bone in the stroma of some soft tissue tumours or even in epithelial cancers, e.g., pilomatrixoma. Notably, in some cancers, it is accepted as a marker of poor prognosis. 

Modern, robust genomic procedures can gently trace transcriptional differences between normal fibroblasts and CAFs. The latter usually present upregulated expression of IL-6 (frequently accompanied by upregulation of IL-8) [20,83]. Very similar findings were confirmed across several types of tumours (Figure 2, Table 3), indicating a general role of IL-6 in cancer biology.

### 5.3. Local and Systemic Effect of IL-6 in Cancer Progression

Factors of paracrine signalling participating in the crosstalk between cancerous and non-malignant cells of the cancer ecosystem profoundly influence the biological behaviour of the tumour [20]. Abundant production of IL-6 by CAFs and other cell types (e.g., adipocytes in breast cancer) in different types of tumours indicates the importance of this factor in cancer cell biology. IL-6 stimulates cancer cell proliferation [93] and epithelial-to-mesenchymal transition [92]. The experimental blockade of IL-6 with the simultaneous inhibition of IL-8 significantly attenuated the invasiveness of cancer cells in vitro [94,95]. The activation of STAT3, JAK/STAT, mTOR, sonic hedgehog and nuclear factor κ B (NFκB) signalling is important for the IL-6 effect on cancer cells and supports the metastatic spreading of malignant disease [96]. The role of IL-6 in cooperation with IL-8 in neovascularisation and thus in the progression of cancer was also confirmed [97]. As demonstrated in Figure 2 and extensively discussed by Lacina and co-workers [20], cancer cells, including the cells of CMM, also produce IL-6. The combination of the paracrine and autocrine routes of production of this cytokine and their complex regulation influencing the CMM cell biology must therefore be expected. 

Factors of the intercellular crosstalk from the cancer site can also cross the capillary wall and thus enter systemic blood circulation. Consequently, these bioactive molecules can be detected in the blood serum of cancer patients [98]. This observation suggests that these molecules might serve as biomarkers and can be potentially used to estimate the progression of the disease. However, problems might come from the specificity of these findings. Moreover, the general health status of the patients must be carefully reflected, because, for example, even a mild respiratory infection before the examination can completely change the serum profile. These factors, produced by the cancer ecosystem and transported by circulation, seem to participate in shaping the premetastatic tissue landscape, a safe niche serving as a suitable cradle for cancer cell homing and later development of metastases, as was demonstrated in the case of breast cancer and malignant melanoma [99,100]. 

Finally, high concentrations of IL-6, IL-10 and TNF-α in the serum can even predict the mortality of patients with an advanced stage of malignant disease [101]. 

Cancer patients frequently die in the terminal, therapy-refractive stage of the disease due to cancer cachexia and wasting. This process seems to be strongly influenced by IL-6 and TNF-α, which affect adipocytes, hepatocytes and striated muscle fibres, where both factors induce skeletal muscle atrophy, lipolysis, the “browning” of white adipocytes and ketogenesis in the liver [20,102,103]. It seems that there is a direct association between the high level of IL-6 produced by the malignant tissue, low skeletal muscle mass, and the survival of the patient [104]. In addition to these severe metabolic problems, IL-6 can cross the blood–brain barrier, where it is recognised by groups of hypothalamic and hippocampal neurons controlling food intake and causing depression [105,106]. A high level of IL-6 even correlates with an increased risk of suicide [107]. The combination of metabolic and central nervous system-related issues seems to be fatal in the terminal stage of the disease when anti-cancer therapy has failed.

### 5.4. Summary of the Role of IL-6 and Cancer

IL-6 represents an important factor of intercellular communication in the cancer cell niche. It also participates in cancer progression, including formation of the premetastatic niche and the process of metastatic dissemination itself. IL-6 has a remarkable systemic effect, culminating, by the failure of metabolism, in severe psychological and mental problems, and finally leading to the death of the cancer patient.

## 6. COVID-19

### 6.1. Covid-19 and IL-6

In contrast to the slow rate of progression of ageing and cancer, the course of acute infectious diseases is associated with an uncontrolled and excessive flare of inflammation. Surprisingly, many molecular players of these clinically distinct conditions remain identical. This offers a useful insight into the regulation of the involved mechanisms. 

COVID-19 is a transmissible respiratory disease caused by coronavirus SARS-CoV-2. The majority of infected persons are, fortunately, asymptomatic, or their symptoms are only mild. Unfortunately, some of the patients develop severe pneumonia accompanied by a risk of damage to other organs such as the liver, heart, digestive system, brain, etc. This severe progression leads to acute respiratory distress syndrome (ARDS), and the illness may result in the failure of respiration and the death of the patient [108,109]. 

COVID-19 is usually accompanied by an elevation of numerous bioactive factors such as IL-1β, TNF-α, IL-2, IL-7, IL-8, IL-9, IL-17 G-CSF, interferon (IFN)-γ, XXC-10, CCL-2 CCL-3, CCL-4, and especially IL-6, which is produced predominantly by macrophages [110,111]. The severe and frequently fatal character of the disease is characterised by a high level of IL-6 and CRP in the blood or plasma of the patients [112,113,114,115]. IL-6, in collaboration with other factors, influences the endothelial cells of lung capillaries, increasing their permeability for serum proteins and improving the transmigration of inflammatory cells [116]. Interestingly, similar findings were noted in earlier serious coronavirus outbreaks, severe acute respiratory syndrome (SARS) and middle east respiratory syndrome (MERS). Both conditions were associated with a severe complication: cytokine storm [117]. This finding demonstrates similarity across serious coronaviral infections. Another well-known respiratory infection, influenza, underlines the role of IL-6 in late immune problems in the patients suffering from these infections. This immune dysregulation can be described as cytokine storm/cytokine released syndrome, where cells such as Tregs, decreasing the level of inflammation, are also reduced. The leading role of IL-6 in this process was also demonstrated in COVID-19. To be characterised as causal for cytokine storm, it should meet the following criteria: 1. rapid and extensive viral replication; 2. infection of airways or alveolar cells; 3. delayed IFN-γ response; 4. monocyte–macrophage and neutrophil accumulation [117]. These conditions are sufficiently accomplished in the COVID-19 disease.

### 6.2. Summary of the Role of IL-6 in COVID-19

IL-6 plays a fundamental role in the advanced stage of COVID-19, where it is associated with the initiation and progression of cytokine storm, which frequently has fatal consequences for the infected person.

## 7. Targeting the IL-6/IL-6R/gp130-Dependent Signalling

As exemplified above, IL-6 signalling is very important in ageing-related disorders, cancer, and severe viral diseases such as SARS, MERS and COVID-19. From this aspect, therapeutic targeting of the IL-6-dependent axis may be vitally important for the treatment of these diseases. The IL-6 pathway-regulating agents can be classified, concerning the biotechnology of their manufacturing, as antibodies and small-molecule inhibitors. 

### 7.1. Antibodies

**Figure 4:**
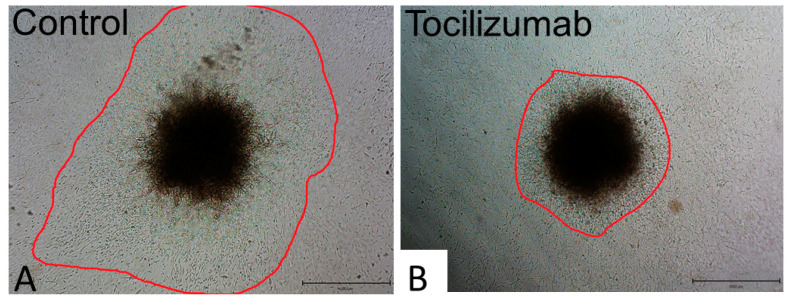
Migration of G361 melanoma cells from spheroids. G361 melanoma cells migrate from the heterogeneous spheres constructed from G361 melanoma cells and juvenile fibroblasts in 3D collagen gels without (**A**) and after tocilizumab application (**B**). Migration of melanoma cells was strongly reduced by the therapeutic humanised monoclonal antibody. Bar is 1 mm.

The prominent representatives of antibodies targeted to IL-6, IL-6R and gp130 are summarised in Table 4. These antibodies are predominantly used for therapy in autoimmune diseases such as rheumatoid/psoriatic arthritis, but their employment as therapeutics for certain tumours is also approved (Table 4). The in vitro anti-migratory effect of some of these antibodies such as tocilizumab suggests the possible employment of IL-6–IL-6R targeting as in migrastatic drugs [118,119] (Figure 4). Unfortunately, the therapeutic effect of migrastatics in anti-cancer treatment is much lower than previously anticipated [120,121]. Perhaps migrastatics in combination with the targeting of other signalling cascades could be more promising. The combination of anti-IL-6 and anti-IL-8 targeting seems to be useful [94,95]. The combination of in vitro and bioinformatic approaches demonstrated that a simultaneous blockade of bascular endothelial growth factor A (VEGF-A) and milk fat globule epidermal growth factor –E8 (MFGE-8) signalling could offer satisfactory results [83]. As summarised by Johnson and co-workers [121], the combination of targeting the IL-6 axis with therapy influencing immune checkpoints can be introduced into clinical practice. 

New data have also demonstrated that antibodies targeting IL-6/IL-6R/gp130 such as tocilizumab, siltuximab and clazakizumab could be employed for the therapy of COVID-19 [122,123,124,125], as was also recommended by the National Institute of Health (NIH COVID-19 Treatment Guidelines, 2020) [126]. The testing of other therapeutic antibodies influencing IL-6 signalling for the treatment of COVID-19 can be expected. 

### 7.2. Natural and Synthetic Small Molecules as IL-6 Receptor Complex Inhibitors

#### 7.2.1. Oestrogen Analogues—Experimental Drugs for Inhibition of IL-6 Signalling

**Figure 5:**
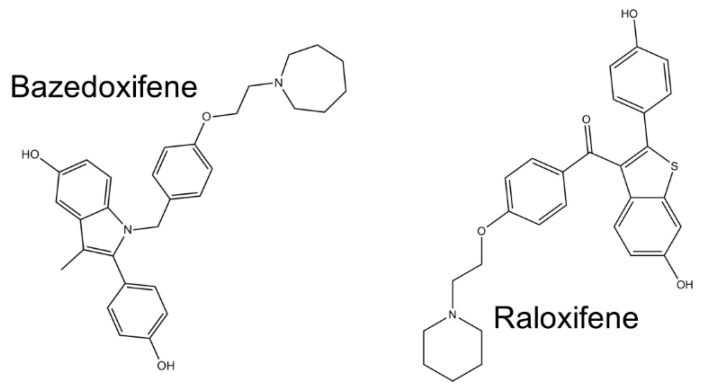
Structure of synthetic oestrogen analogues bazedoxifene and raloxifene.

An interesting molecule with a documented potential to block IL-6R is a synthetic analogue of oestrogens, bazedoxifene. This clinically available drug was designed and later approved for the therapy of postmenopausal osteoporosis [127]. Another substance with a very similar structure was prepared for the same purpose: raloxifene [128] (Figure 5). These therapeutics are also able to interact with gp130 and thus inhibit docking of IL-6 to its receptor [129,130]. Because of their low price and minimal adverse effects, these substances were tested for the therapy of some malignant tumours such as rhabdomyosarcoma [129], head and neck cancer [131], adenocarcinoma of the pancreas [132,133], colorectal cancer [134], and hepatocellular carcinoma [135]. They were also proposed for the treatment of cytokine storm in patients suffering from COVID-19 [136,137,138]. Moreover, bazedoxifene also reduces the replication of SARS-CoV-2 in susceptive cells [139].

#### 7.2.2. Other Small Molecules—Experimental Drugs

Recently, it was demonstrated that targeting the IL-6 receptor by monoclonal antibodies is a promising therapy for a number of diseases associated with increased inflammation. However, monoclonal antibodies have some limitations (high cost, invasive route of administration, and appreciable rate of immunogenicity) to their clinical benefit [140]. Therefore, the development of low-molecular weight inhibitors is highly demanded for their superiority in oral absorption, low toxicity, and low antigenicity. Despite the immense importance of this task and the invested efforts, the IL-6 axis-influencing compounds are only few [141,142,143,144,145,146,147,148,149]. 

**Figure 6:**
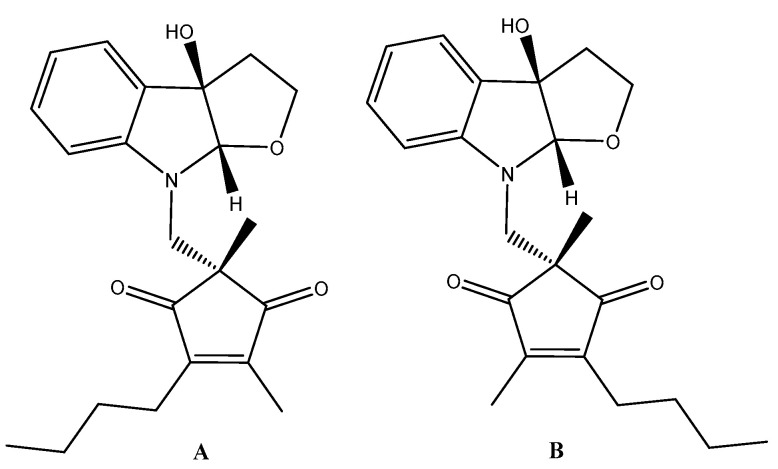
Madindoline regioisomers (**A**) and (**B**).

For example, natural compounds (madindolines A and B) (Figure 6) produced by Streptomyces sp. displayed vigorous inhibition activity against the growth rate of IL-6-dependent cell lines [141,145]. It was observed that the addition of higher IL-6 levels repressed this phenomenon, and the growth rate of IL-6-independent lines was not affected, implying that these compounds could target the IL-6 receptor complex. However, subsequent studies showed that the effect of madindoline is based on its binding to gp130 [142]. Madindoline A did not affect osteoclast formation controlled by the heterodimer type of gp130 (LIF-induced) or cAMP (IL-1), but, in this case, the homodimer types of gp130 (induced by IL-6 and Il-11) were found to be significantly efficient. 

In the C3H-HeJ mouse model (lipopolysaccharide-insensitive), secretion of serum amyloid induced by IL-6 was inhibited by madindoline A in a dose-dependent manner. However, the secretion of serum amyloid induced by lipopolysaccharide-sensitive C3H-HeN mice) was not reduced by madindoline A [144]. These facts also ignited the development of a novel synthetic madindoline analogue. For example, Yamamoto and co-workers [146] prepared a library of candidate structures and tested their effect on the growth of 7TDI cells (IL-6-dependent cell line). These authors proposed that hydrophobic substitution by acyl chains can sometimes improve madindoline inhibition activity. 

**Figure 7:**
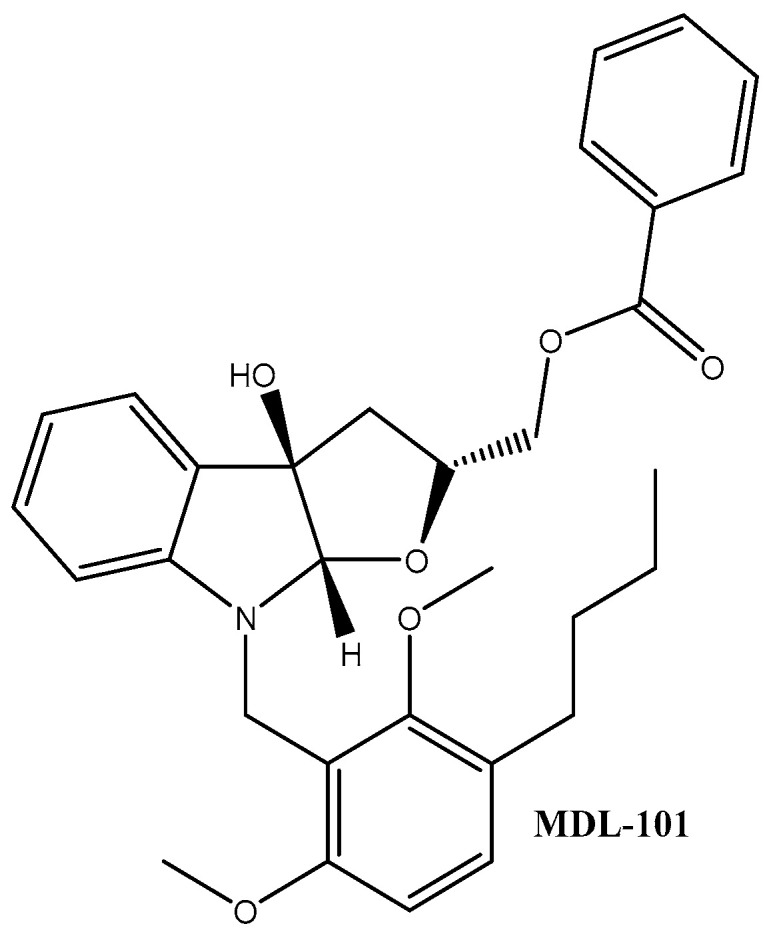
MDL-101 derivative of madindoline.

A promising therapeutic application of madindoline analogues such as MDL-101 (Figure 7) for the treatment of neurodegenerative diseases was also demonstrated by Aqel et al. [147]. These compounds can also interfere via IL-17 production (induced by STAT 3 signalling) in myelin-specific CD4 T lymphocytes in a dose-dependent manner.

**Figure 8:**
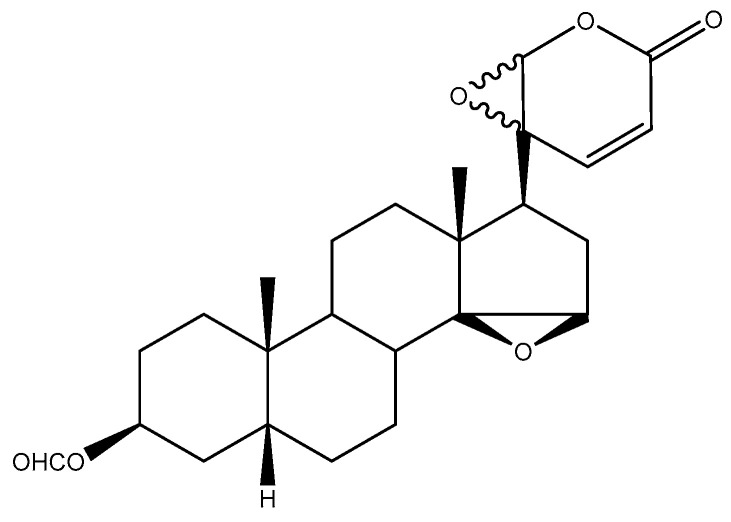
20S,21-Epoxy-resibufogenin-3-formate (ERBF) inhibitor.

Other compounds targeting the IL-6 receptor are bufadienolide derivatives. These natural anti-cancer compounds are isolated from a Chinese toad skin extract—the Ch’an Su drug [150]. It contains active components such as 20S,21-epoxy-resibufogenin-3-formate (ERBF, Figure 8) [142]. This compound did not affect IL-2-, IL-3- and IL-5-dependent cell growth. However, in the case of IL-6-dependent cell lines, the effect of this molecule was notable. In a co-culture of osteoblasts and bone marrow cells, similar to madindoline A, the repression of IL-6 induced osteoblast formation. The effect of substances such as LIF and 1-25(OH)2D3 vitamin was not compromised. Enomoto and colleagues [143] demonstrated that the mechanism of its effect on the IL-6 signalling axis is based on the blockade of IL-6 interaction with its receptor. This finding is substantial because ERBF could treat pathologies such as cancer cachexia, which is associated with IL-6 overactivity. This was demonstrated in an experimental model of colon cancer-induced cachexia. ERBF markedly inhibited body weight loss, but, unfortunately, did not affect tumour growth. 

The relationship between the structure of bufadienolide derivatives and their inhibition activity was studied using IL-6-dependent and independent MH-60 cell lines [143]. Both epoxides at the C-14, C-15 and C-20, C-21 positions in the structure are required to exhibit the inhibitory activity, and the C-16 position must be unsubstituted. The introduction of aliphatic organic acid in the C-3 position increased the inhibition activity in IL-6-dependent cells. This inhibition activity decreased according to the increase in the carbon chains of fatty acids at the C-3 position, such as propionate, butyrate and isobutyrate, whereas a carbonyl group at the C-3 position exhibited cytotoxic activity for both types of MH-60 cells. The above facts inspired Kino et al. [145] to study the effects of bufadienolide derivatives such as TB-2-081 (3-O-formyl-20R,21 epoxyresibufogenin) on the IL-6 signalling in the hepatocyte cell lines. As expected, the authors observed a reduced expression of IL-6-controlled genes (e.g., α1-antichymotrypsin, α1-acid glycoprotein, α2-macroglobulin, and β-fibrinogen) and low secretion of C-reactive protein. Nevertheless, because IL-11-induced α1-antichymotrypsin expression was also repressed, this implies that the effect of the tested compounds is based on the inhibition of gp130 and not directly on the level of IL-6R.

**Figure 9:**
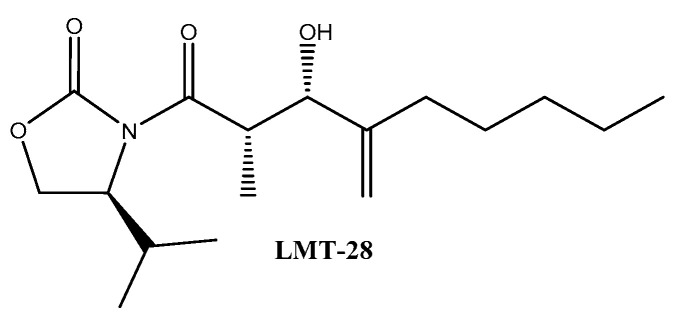
LMT-28 inhibitor.

Another interesting inhibitor targeting gp130, LMT-28 (Figure 9), was designed by Hong and co-workers [148]. It interacts with gp130 and subsequently reduces the affinity of the receptor complex for the binding of available IL-6. In agreement with this mechanistic explanation, this leads to a reduction in STAT3 phosphorylation, stimulated by IL-6 in permissive cells. This observation was further confirmed in a mouse model. 

In general terms, certain structural motives (e.g., bufadienolide and madindoline derivatives) are suitable for targeting IL-6 receptors and the suppression of IL-6 pathway signalling activity. They were shown to display low toxicity. Consequent studies performed in vitro and in vivo offered some therapeutic potential, for example, for the treatment of inflammatory, neurodegenerative, and also oncological diseases. Their biological effects are summarised in Supplementary Table S1. Nevertheless, to progress towards their clinical application, and a more in-depth understanding of the relationship between their molecular structure and biological effect must first be achieved. Notably, it is crucial to improve their in vivo delivery to suitable cells.

Other examples of synthetic/natural small-molecule inhibitors that affect IL-6 production, docking and signalling, including the description of the molecular mechanism, are excellently provided in a recent review by Kaur and co-workers [151]. 

**Figure 10:**
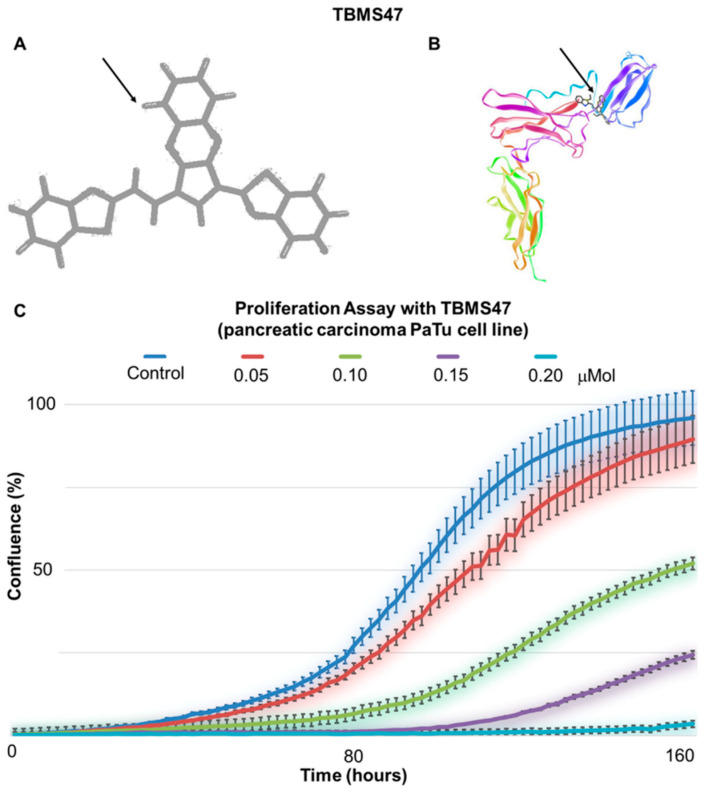
Inhibitor TBMS47 (**A**—structure, **B**—model). Chemical structure of experimental substance TBMS47 (arrow) was designed to interact with IL-6R and its docking to the binding site of IL-6R recognizing IL-6. (**C**) TBMS47 inhibits in vitro proliferation of PaTu cells from pancreatic adenocarcinoma (represented here as Confluence %) in a concentration-dependent manner measured using Incucyte instrumentation (each line represents six technical replicates; error bars represent standard deviation of six wells).

As an example, we show the efficiency of the experimental substance TBMS47, developed in our laboratory. The substance was designed to be active in micromolar concentrations, such as is requested of modern low-molecular weight anticancer drugs [152]. However, the therapeutic concentration in clinics could has not yet been estimated. TBMS47 recognises the biding site of IL-6R that blocks the interaction between IL-6 and IL-6R. In vitro application of this molecule has a significant effect on the growth of melanoma cells, and the effect is concentration dependent (Figure 10).

Until now, numerous studies focused on the association of the chemical structure of the inhibitor and its biological effect. However, several essential issues still remain open. For example, the inhibition effect of ERBF is dependent on the blocking of the interaction of IL-6 with its receptor. Other bufadienolide derivatives (e.g., TB-2-081)—against expectation—inhibit the interaction of gp130 with the complex of IL-6 and IL-6R [145]. It is well known that some types of cancer are associated with the mutation of proteins of the IL-6 axis that can significantly influence IL-6 signalling [13,153,154]. The strict requirement to employ a distinct inhibitor to receive a correct biological response is still valid. Robust and mechanistically clear studies of the IL-6 signalling cascade and its specific inhibition are highly desirable before clinical application of the novel inhibitors.

In the case of small molecules, low solubility remains one of the greatest issues. In particular, poorly soluble ones suffer from insufficient selectivity for the target organs and tissues, and their half-life in the blood is short. Currently, numerous suitable drug delivery systems for these types of compounds are being developed or are already available, for example, cyclodextrins, silica nanoparticles, and liposomes [155,156,157]. Notably, these systems can be successfully used for drug transport across the blood–brain barrier. This can significantly enhance the therapeutic potential of these small IL-6 receptor inhibitors for the treatment of brain tumours or neurological diseases.

## 8. Direct Targeting of CAFs

In the case of malignant tumours with a prominent role in CAFs, therapy targeting different molecules in these cells is in the phase of clinical trials, as summarised in a recent review by Sahai and co-workers [71]. Suitable targets are FGF receptor (FGFR), hedgehog, TGF-β, CXC-chemokine receptor 4 (CXCR-4), RHO kinase (ROCK), focal adhesion kinase (FAK), lysyl oxidase-like 2 (LOXL-2), connective tissue growth factor (GTF), hyaluronic acid, and FAP. The targeting of CAFs using a synthetic antibody analogue (iBody) directed to FAP by a sensitive substrate appears to be rather promising [158,159]. Despite the prominent role of IL-6 in tumour biology, this aspect of stromal biology seems to have been somewhat neglected until now. Nevertheless, it is too early to evaluate the therapeutic relevance of these approaches and determine their position among other recently available treatment options. 

## 9. Concluding Remarks

IL-6 is a multifaceted cytokine with a remarkable role in the initiation of inflammation and immune response. On the other hand, the failure of regulation and increased levels of this cytokine in a patient’s body are influenced by ageing, cancer progression and fatal complications of serious viral infections. The high level of IL-6 and abnormal activation of the IL-6–IL-6R axis are associated with the severe progression of disease and may be responsible for the failure of therapy and, eventually, fatal complications. A detailed understanding of the biology of IL-6, the IL-6R receptor and its signalling axis can bring new information essential for the amelioration of the problems of ageing and offer an efficient therapy for malignancies and viral infections. A panel of therapeutic antibodies influencing IL-6 signalling is available, but their use has various biological and economic limitations. Another potential modality is represented by the class of small-molecule inhibitors. Nevertheless, in-depth knowledge of the biology of IL-6 signalling along with the precise determination of the relationship between the inhibitor’s chemical structure and the IL-6–IL-6R complex are prerequisites for its rapid addition to the therapeutic arsenal. 

## Figures and Tables

**Table 1:** Synonyms for interleukin-6 (IL-6).

Name	Author
Interferon β-2	Zilberstein et al., 1986 [2]
26K factor	Haegeman et al., 1986 [3]
B-cell stimulatory factor	Hirano et al., 1985 [4]
Hybridoma growth factor	Brakenhoff et al., 1987 [5]
Plasmacytoma growth factor	Nordan et al., 1987 [6]
Hepatocyte stimulatory factor	Gauldie et al., 1987 [7]
Haematopoietic factor	Ikebuchi et al., 1987 [8]
Cytotoxic T-cell differentiation factor	Takai et al., 1988 [9]

**Table 2:** Examples of cells producing IL-6.

Type of cell	Author
Keratinocyte	Groeger and Meyle, 2019 [10]
Enterocyte	Pritts et al., 2002 [11]
Urothelium	Uehling et al., 1999 [12]
Hepatocyte	Schmidt-Arras and Rose-John, 2016 [13]
Pneumocyte and bronchial epithelial cell	Cheung, 2005 [14]
Smooth muscle	Kyotani et al., 2019 [15]
Skeletal muscle	Barbalho et al., 2020 [16]
Osteoblast	Kovács et al., 2019 [17]
Adipocyte	Xie et al., 2019 [18]
Macrophage	Shapouri-Moghaddam et al., 2018 [19]
Neuron	Shapouri-Moghaddam et al., 2018 [19]

**Table 3:** Examples of production of IL-6 by CAFs in different types of cancer and its effect on cancer.

Type of Cell	Effect on Tumour Growth and Spreading	Author
Prostate	+	Heneberg, 2016 [84]
Adenocarcinoma of pancreas	+	Heneberg, 2016 [84]
Liver	+	Li et al., 2019 [85]
Colorectal	+	Nagasaki et al., 2014 [86]
Stomach	+	Wu et al., 2017 [87]
Lung	+	Wang et al., 2017 [88]
Head and neck squamous cell carcinoma	+	Plzák et al., 2019 [83]
Basal cell carcinoma of skin	+	Omland et al., 2017 [89]
Squamous cell carcinoma of skin	+	Depner et al., 2014 [90]
Cutaneous malignant melanoma	+	Jobe et al., 2018 [91]
Urinary bladder	+	Goulet et al., 2019 [92]

**Table 4:** Examples of antibodies designed to target IL-6, IL-6R and gp130.

Antibody	Target	Main Application	Producer
Siltuximab *	IL-6	Renal + prostate cancer Castleman’s disease COVID-19	EUSA Pharma
Sirukumab +	IL-6	Rheumatoid arthritis COVID-19	Janssen Biotech
Olokizumab +	IL-6	Rheumatoid arthritis	R-Pharm Group
Clazakizumab +	IL-6	Psoriatic arthritis COVID-19	Bristol Myers Squibb and Alder Biopharmaceuticals
Elsilimomab +	IL-6	Lymphoma Myeloma	Diaclone
Tocilizumab *	IL-6R	Rheumatoid arthritis Multiple myeloma Prostate cancer COVID-19	Hoffmann-La Roche and Chugai
Sarilumab *	Gp130	Rheumatoid arthritis	Regeneron Pharmaceuticals and Sanofi

* used in clinical practice, + experimental or under clinical trial.

## Supplementary Materials

Supplementary materials can be found at https://www.mdpi.com/1422-0067/21/21/7937/s1.

## Abbreviations

ASA	Acetylsalicylic acid
ARDS	Acute respiratory distress syndrome
BCC	Basal cell carcinoma
BCCF	Basal cell carcinoma-associated fibroblasts
BDDF	Brain-derived neurotrophic factor
CNTF	Ciliary neurotrophic factor
CAFs	Cancer-associated fibroblasts
CLC	Cardiotrophin-like cytokine
COX	Cyclooxygenase
CRP	C-reactive protein
CXCR-4	CXC-chemokine receptor 4
DAPI	4’,6-Diamidino-2-phenylindole
DF	Dermal fibroblast
ERBF	20S,21-Epoxy-resibufogenin-3-formate
FAK	Focal adhesion kinase
FAP	Fibroblast-activating protein
FGF	Fibroblast growth factor
FGFR	Fibroblast growth factor receptor
GTF	Connective tissue growth factor
G-CSF	Granulocyte colony-stimulating factor
Gp130	Glycoprotein 130
IFN	Interferon
IGF-1	Insulin growth factor-1
IL	Interleukin
IL-6R	Receptor for IL-6
LIF	Leukaemia inhibitory factor
LOXL-2	Lysyl oxidase-like 2
MFGE8	Milk fat globule epiderma drowth factor E8
MERS	Middle east respiratory syndrome
NFκB	Nuclear factor κ B
NK cells	Nature killer cells
PDGF	Platelet-derived growth factor
ROCK	RHO kinase
ROS	Reactive oxygen species
SARS	Severe acute respiratory syndrome
SASP	Senescence-associated secretory phenotype
SCC	Squamous cell carcinoma
SCCF	Squamous cell carcinoma-associated fibroblasts
sHH	Sonic hedgehog
SMA	α-Smooth muscle actin
Treg	Regulatory T lymphocytes
TGF-β	Transforming growth factor β
TNF	Tumour necrosis factor
VEGFA	Vascular endothelial growth factor A
