# Supplementary material for: Interleukin-6: Molecule in the Intersection of Cancer, Ageing and COVID-19
Source: Int J Mol Sci. 2020 Oct 26;21(21):7937. doi: 10.3390/ijms21217937 (PMC7662856; doi:10.3390/ijms21217937)
Supplement: Supplementary file 1 [file ijms-21-07937-s001.pdf]

Supplementary Table 1. Inhibitors of IL-6 receptor in the biological system.

Compound	Inhibition of IL-6 receptor *in vitro*	
	Effect on the cell proliferation, inducing factor-cell type	
	IL-6 and L-6 depended	IL-6 independent
madindoline A(Hayashi, Kim et al. 1996)	↓MH60, IC_{50}=8 μmol/l	**0**IL-6-(B16, SC- 115 P388/ADM, CPAE, HUVEC and HL60)
madindoline B	↓MH60, IC_{50}=30 μmol/l	
madindoline A(Hayashi, Rho et al. 2002) /(10-100μmol/l)	↓MH 60, parallel rightward shift of dose–response curves to IL-6 (2–8 ng/ml)	**0**IL2-CTLL-2, **0**IL-3-Baf3 and **0**TNF-α-L929
ERBF (15umol) (Hayashi, Rho et al. 2002)	↓MH-60, parallel rightward shift of dose–response curves to IL-6 (0.03 to 10 ng/ml)	**0**IL-2-(CTLL-2 and MH-60[a]), **0**IL-3-(Baf3 and Baf3/GCSFR-gp130) and **0**GCSF-Baf3/GCSFR-gp130 **0**TNF-α-L929
(Enomoto, Rho et al. 2004) ERBF	↓MH-60, IC_{50}=5.3 μmol/l, parallel rightward shift of dose–response curve to IL-6	
LMT-28(Hong, Choi et al. 2015)	↓HEPG2, IC_{50}=5.9 μmol/l	**0**LIF-HEPG2 and ↓IL-11-HEPG2
	Effect on the cellular phenotype, factor (targeted protein and phenomena) cell type	
madindoline A(Hayashi, Rho et al. 2002)	↓IL6-(differentiation to macrophage-like cells) M1 cells and ↓IL6-(pSTAT3) Baf3-GCSFRgp130 Cells	
MDL-101(Aqel, Kraus et al. 2019)	↓IL6 (IL-17, pSTAT-3 expression and Th17 development) myelin-specific CD4 T	
(Hayashi, Rho et al. 2002) ERBF	**0**IL-4-(expression of CD23) U937, **0**IL-8-(chemotaxis) PMNLs and **0**NGF-(neuronal differentiation) PC-12	
TB-2-082(Kino, Boos et al. 2007)	↓IL-6 (AACT mRNA and pSTAT3) HepG2, ↓IL-6 (mRNA of AGP, α2-macro, and β-fibrinogen and CRP secretion) rat primary hepatocytes, **0**TNF-α-(MAT2A mRNA)HepG2, ↓IL-11 and ↓OSM-(AACT mRNA) HepG2	
LMT-28(Hong, Choi et al. 2015)	↓IL-6(p STAT3, gp130, and JAK2 protein, TNF-α, proliferation) TF-1, and↓IL-6(pSTAT3) HepG2	
Osteoclast formation (coculture of osteoblastic cells and bone marrow cells)		
madindoline A (Hayashi, Rho et al. 2002)	↓IL-6, ↓IL-11, **0**LIF and **0**1α,$25(OH)_2D_3$	
ERBF (Hayashi, Rho et al. 2002, Enomoto, Rho et al. 2004)	↓IL-6, **0**IL-11, **0**LIF, and **0**1α,$25(OH)_2D_3$	
	Mice model	
madindoline A (60 mg/kg per day; oral) (Hayashi, Rho et al. 2002)	OVX Mice	↓reduction of bone mass and ↑serum Ca^{2+} level

madindoline A (10, or 60mg/kg; oral) (Hayashi, Rho et al. 2002)	LPS-insensitive C3H-HeJ mice with IL-6 (1 µg per mouse)	↓SAA secretion, dose dependently
MDL-101 (in splenocytes) (Aqel, Kraus et al. 2019)	naive B10PL mice with encephalitogenic splenocytes	↓myelin-specific CD4 T cells
	SJL/J mice with encephalitogenic splenocytes	↓EA development
MDL-101 (50 mg/kg x7/ day; injected) (Aqel, Kraus et al. 2019)		no effect
(Enomoto, Rho et al. 2004) (10, or 30 mg/kg; oral)	colon-26-bearing BALB/c mice	↓reduction of carcass weight, dose dependently
LMT-28 (0.4 or 0.8 mg/kg; oral) (Hong, Choi et al. 2015)	C57BL/6 mice	↓TNF-α, dose dependently
LMT-28 (0.25 mg/kg; oral) (Hong, Choi et al. 2015)	DBA/1J mice with CIA	↓Arthritis scores (COMP by 50%, SAP by 55% anti-CII IgG by 62%)
LMT-28 (1 mg/kg; oral) (Hong, Choi et al. 2015)	Male BALB/c mice with cerulein induced pancreatis	↓IL-1β, ↓TNF-α, ↓IL-6, ↓edema, ↓inflammatory cell infiltration and ↓necrosis

[a]MH-60 with induced independence on IL-6

B16 (melanoma), CPAE (calf pulmonary artery endothelial cells), HUVEC (human umbilical vein endothelial cells) HL60 (human leukemia), HEPG2 (human liver carcinoma), L929 (mouse fibrosarcoma), P388/ADM (adriamycin-resistant leukemia), PC-12 (Rat phenochromocytoma), PMNLs (Human polymorphonuclear leukocytes). SC-115 (hormone-dependent Shionogi carcinoma), U937 (human myeloid leukemia)

01α,25(OH)$_2$D$_3$ (1a,25-dihydroxyvitamin D3), AACT (a1-antichymotrypsin), AGP(α1-acid glycoprotein), α2-macro(α2-macroglobulin), CII (type II collagen), CIA (collagen-induced arthritis), COMP (cartilage oligomeric matrix protein), CRP(C-reactive protein), LIF (leukemia inhibitory factor), MAT2A (methionine adenosyltransferase 2A), NGF (nerve growth factor), OSM (oncostatin M), SAA (Serum Amyloid A), SJL (Swiss Jackson Laboratory), SAP (serum amyloid P),

0=no effect, ↓=reduction of activity, expression of controlled protein, or phenomenon, ↑=increase of activity, expression of controlled protein, or phenomenon
